# The Toxicity of Selected Trace Metals to *Lecane inermis* Rotifers Isolated from Activated Sludge

**DOI:** 10.1007/s00128-013-1062-z

**Published:** 2013-07-18

**Authors:** Beata Klimek, Edyta Fiałkowska, Wioleta Kocerba-Soroka, Janusz Fyda, Mateusz Sobczyk, Agnieszka Pajdak-Stós

**Affiliations:** Institute of Environmental Sciences, Jagiellonian University, Gronostajowa 7, 30-387 Kraków, Poland

**Keywords:** Trace metal, LC50 value, Rotifers, Activated sludge

## Abstract

The aim of the study was to assess the toxicity of a range of trace metals to the rotifer *Lecane inermis*, a species tested as a potential biological tool to control activated sludge bulking caused by overgrowth of filamentous bacteria in wastewater treatment plants. LC50 values (concentration lethal to 50 % of individuals, mg dm^−3^) were ranked in the following order: Cu < Al < Fe < Zn < Sn < Mn. *L. inermis* apparently is more sensitive to metals than other aquatic species widely used as model organisms in ecotoxicological testing, making it potentially useful for quick ecotoxicological tests.

Municipal and industrial sewage is a heterogeneous mixture of organic and inorganic compounds, some of which are potentially toxic to organisms living in activated sludge in biological wastewater treatment plants. Sewage purification processes depend strongly on the condition of the activated sludge, which can be disturbed under high loads of chemicals. Heavy metals are chemical elements with density above 5 g cm^−3^ (Nies 1999). They are not biodegradable and are difficult to neutralise. Their presence in wastewater is of great environmental concern. They also compromise biological activity in activated sludge, adversely affecting biological wastewater treatment processes (Sin et al. 2000, Chipasa 2003). Some heavy metals are essential and required by organisms as nutrients, but at higher concentrations both essential and non-essential metals can exert toxic effects on organisms (Chodak et al. 2013).

The heavy metal content of wastewater from industry and in municipal sewage depends on many local factors such as the types of industry in the region, people’s lifestyle habits, and public awareness of the environmental impact of careless disposal of waste. The quality of water purification is improving globally but water quality remains a major environmental concern. Knowledge of the effects of metals on activated sludge organisms is needed for proper assessment of acceptable metal concentrations in wastewater and for predicting problems with purification processes. As the majority of laboratories at wastewater treatment plants do not have advanced analytical equipment, simple biological tests offer a reasonable alternative for analysing the potential toxicity of wastewater.

Rotifers are one of the group of organisms composed of activated sludge biocenoses (Fiałkowska and Pajdak-Stós 2008). They feed on dispersed bacteria bound with sludge flocs as well as suspended biomass, reducing both the bacteria and the density of suspended particles, thereby playing an important role in reducing excessive sludge generation in wastewater treatment (Lapinski and Tunnacliffe 2003). As recently discovered, *Lecane inermis* rotifers are able to control the overgrowth of troublesome filamentous bacteria such as *Microthrix parvicella* (Fiałkowska and Pajdak-Stós 2008). To overcome activated sludge bulking and foaming, iron and aluminium chlorides are widely used, commercially available as PIX and PAX (Geneja 2008). These substances improve the settlement characteristics of sludge, but with increased dosage of those products in activated sludge, an increase of free bacteria and a decrease of protozoan activity are observed (Roels et al. 2002).

Thanks to a number of promising characteristics, rotifers play an increasing role in assessing the effects of environmental contaminants on aquatic ecosystems (Dahms et al. 2011). Commercially available standardised toxicological tests like Rotoxkit F™ are based on the marine rotifer *Brachionus* spp. (Mankiewicz-Boczek et al. 2008), which is not found in activated sludge. Here we propose the use of *Lecane inermis* instead. It is a rotifer species normally found in activated sludge. We measured the 24 h acute toxicity of six heavy metals—aluminium, copper, iron, manganese, tin and zinc—in order to estimate their LC50 values, that is, the metal concentrations causing 50 % rotifer mortality.

## Materials and Methods

*Lecane inermis* (Monogonta), a rotifer species commonly occurring in wastewater treatment plants, was used in the experiments. Clonal population Lk3 was obtained from a single individual isolated from a treatment plant in southern Poland. The culture was maintained continuously in our laboratory for approximately 1 year in darkness at 20°C (Sanyo Versatile Environmental Test Chambers).

We performed range-finding tests using five concentrations plus controls for each toxicant. Test concentrations represented a logarithmic series and ranged from 0.001 to 10 g dm^−3^ for each salt employed: AlCl_3_, CuSO_4_·5H_2_O, FeCl_3_·6H_2_O, MnCl_2_·4H_2_O, SnCl_2_·2H_2_O and Zn(NO_3_)_2_·6H_2_O (POCh Poland). The lowest concentration giving 100 % mortality was taken as the upper limit and additional intermediate concentrations were included in the final test; finally, from three to six concentrations of a given metal salt plus control treatments were applied. The salt solutions were prepared using Żywiec brand mineral water enriched with molasses as medium (Greenland Technology, Poland). The rotifers used in the experiments were cultured from eggs to produce individuals at a similar development stage (age <1 day). Ten rotifers were collected with a micropipette and transferred to separate wells (24-well Cell Wells™, Corning). Then 1 ml of a given solution of metal salt was added. The culture plates were incubated at 20°C and the number of live and dead rotifers was recorded after 24 h exposure. Tests in which control mortality was 5 % or more were excluded from the analyses. Each treatment was applied in four to six replicates. The LC50 values (mg dm^−3^) were calculated from a linear model for each tested element. For each element ${\text{R}}_{\text{adj}}^{ 2}$ was given, that is statistic indicates % of variability in data explained by the model, adjusted for number of degrees of freedom.

## Results and Discussion

The results of the experiment are presented in Table 1. The tested element most toxic to *Lecane inermis* rotifers was copper, with an LC50 value of 0.0250 mg dm^−3^ Cu^2+^ ions. After copper, aluminium and iron were the most toxic, followed by zinc and tin. The toxicity levels of zinc and tin were approximately the same (0.1574 and 0.1618 mg dm^−3^ respectively). The least toxic element was manganese, with an LC50 value of 1.9194 mg dm^−3^.

**Table 1:** Metal toxicity to *Lecane inermis* rotifers

	LC50 value (mg dm^−3^)	${\text{R}}_{\text{adj}}^{ 2}$ (%)	95 % confidence interval (mg dm^−3^)	
			Lower limit	Upper limit
Cu^2+^	0.0250	81.2	0.0205	0.0294
Al^3+^	0.0518	95.8	0.0471	0.0565
Fe^3+^	0.0884	95.6	0.0801	0.0967
Zn^2+^	0.1574	90.0	0.1404	0.1743
Sn^2+^	0.1618	85.9	0.1356	0.1880
Mn^2+^	1.9194	75.8	1.6203	2.2186

Metals differ in their toxicity to organisms, and our results are generally consistent with a number of similar studies conducted on rotifers and other organisms. Others have found copper and zinc to be among the elements most toxic to activated sludge organisms (Nicolau et al. 2005). Significant sources of zinc and copper in wastewater are metal-processing industries, manufacture of paints, plastics and alloys, as well as acidic water from mine drainage (Sin et al. 2000). Copper is rarely found in natural water bodies but is found in polluted environments (Udom et al. 2004). It poses a serious threat to organisms inhabiting activated sludge, and to ecosystems when sludge is used excessively in agriculture or remediation. For example, copper has been shown to be more toxic than zinc to the fungus *Trichoderma atroviride* isolated from sludge (Errasquín and Vázquez 2003), to various species of ciliated protozoa (Madoni et al. 1994), to the sludge worm *Tubifex tubifex* (Rathore and Khangarot 2002), and to some non-aquatic organisms (Chaperon and Sauvé 2007). It is reasonable to take these findings as indicative of a general rule. The LC50 determined for Cu toxicity to *Brachionus calyciflorus* was 0.026 mg dm^−3^ (Preston and Snell 2001), similar to that determined for *Lecane inermis*. The LC50 value determined for zinc toxicity to *Lecane quadridentata* is 0.1231 mg dm^−3^ (Guzmán et al. 2010), similar to LC50 for *Lecane inermis* in our experiment.

Among the metals normally encountered in aquatic systems, aluminium and iron are commonly present in polluted aquatic ecosystems (Shaw et al. 2006). The main sources of increased iron concentrations in the aquatic environment are acidic water from mine drainage, mineral and steel processing and industrial runoff (Guzmán et al. 2010). In *Lecane quadridentata*, Guzmán et al. (2010) gave LC50 values of 0.1572 mg dm^−3^ for aluminium and 0.5390 mg dm^−3^ for iron. A comparison with our results (LC50 of 0.0518 mg dm^−3^ for aluminium and 0.0884 mg dm^−3^ for iron) indicates that *L. inermis* is more sensitive than *L. quadridentata* to both these metals.

Acidic water from mine drainage is also a major source of tin (Sheoran and Sheoran 2006), and the wastewater discharged from the steel industry generally carries high loads of manganese (Xu et al. 2009). Manganese and tin are rarely treated as hazardous pollutants although their toxic effects on aquatic organisms have been reported (Rathore and Khangarot 2002, Pawlik-Skowrońska et al. 1997). In our study the least toxic metal was manganese. The LC50 value for manganese ions was two orders of magnitude higher than for copper, in line with results from experiments on *Tubifex tubifex* (Rathore and Khangarot 2002). To our knowledge the acute toxicity of manganese and tin to rotifers has not been investigated previously.

An important problem for the future is standardisation of experimental conditions to achieve repeatability of results for toxicity assessment. A good example of the importance of experimental conditions comes from two studies on *L. quadridentata*. McDaniel and Snell (1999) gave an LC50 value of 0.046 mg dm^−3^ for cadmium. Pérez-Legaspi and Rico-Martínez (1998) gave an LC50 value of 0.28 mg dm^−3^ for it—more than six times higher. Factors such as water hardness, pH and temperature affect the toxicity of heavy metals to water invertebrates (Gupta et al. 2001, De Schamphelaere et al. 2006), and other factors not controlled in a protocol can play a role. In our previous experiment, conducted in the same laboratory and with the same protocol, we found an LC50 value for aluminium toxicity to *Lecane inermis* much higher than in the current experiment: for aluminium it was as high as 0.012 mg dm^−3^ (Klimek et al. 2013, in press) as compared to the present result, 0.0518 mg dm^−3^.

Ecotoxicological data on even common pollutants are scarce for *Lecane* spp. The most scrupulous ecotoxicological study on three *Lecane* spp. rotifers using a range of different chemicals was done by Pérez-Legaspi and Rico-Martínez (1998). Rotifers are small, widely distributed, easy to cultivate and genetically homozygous, and they reproduce rapidly. These features make them a very good model organism for ecotoxicological testing (Preston and Snell 2001, Dahms et al. 2011). Despite the lack of ecotoxicological data in the literature, *Lecane inermis* presents a promising model organism for quick toxicological testing of wastewater. The main advantage of this species is that it is often found in wastewater treatment plants. Since the use of *L. inermis* to help overcome activated sludge bulking has been proposed recently, this species may become commercially available in the near future (Fiałkowska and Pajdak-Stós 2008).
